# Monolithic processing of a layered flexible robotic actuator film for kinetic electronics

**DOI:** 10.1038/s41598-021-99500-9

**Published:** 2021-10-08

**Authors:** Shiyi Zhang, Joseph Wang, Kenshi Hayashi, Fumihiro Sassa

**Affiliations:** 1 Graduate School of Information Science and Electrical Engineering, Kyushu University, 744 Motooka, Nishi-ku, Fukuoka 819-0395 Japan; 2 Department of Nanoengineering, Center of Wearable Sensors, University of California San Diego, La Jolla, CA USA

**Keywords:** Engineering, Physics

## Abstract

Low-invasive soft robotic techniques can potentially be used for developing next-generation body–machine interfaces. Most soft robots require complicated fabrication processes involving 3D printing and bonding/assembling. In this letter, we describe a monolithic soft microrobot fabrication process for the mass production of soft film robots with a complex structure by simple 2D processing of a robotic actuator film. The 45 µg/mm^2^ lightweight film robot can be driven at a voltage of CMOS compatible 5 V with 0.15 mm^−1^ large curvature changes; it can generate a force 5.7 times greater than its self-weight. In a durability test, actuation could be carried out over 8000 times without degradation. To further demonstrate this technique, three types of film robots with multiple degrees of freedom and a moving illuminator robot were fabricated. This technique can easily integrate various electrical circuits developed in the past to robotic systems and can be used for developing advanced wearable sensing devices; it can be called “Kinetic electronics”.

## Introduction

Advanced assistive devices such as non-invasive sensors, intelligent eyeglasses, and robot suits support disabled and elderly people and expansion of all human activity^1–3^. For example, wearable chemical or biological sensors based on flexible electronics^4–7^ can monitor human health and activity and obtain environmental information about a person’s surroundings^8–11^. A major bottleneck in the application of such devices to humans is the body–machine interface. Mismatches exist between the living body, which is always deforming or growing, and the unmoving rigid machines. The use of soft robots^12,13^ with movement similar to that of living organisms that have a flexible body is a promising approach for solving this problem. Soft robots consist of a soft material body and flexible actuators such as electroactive polymers (EAPs)^14,15^, pneumatic actuators^16–18^, shape memory polymer actuators^19–21^, magnetic actuators^22–24^, light responsive actuators^25,26^, electrothermal actuators^27–29^, and hydrogel actuators^30,31^. They are resistant to mechanical impact and deformation and enable soft creature-like movement^32^. Thus, the use of soft robot techniques is a promising way to develop body-friendly creature-like body–machine interfaces, which can allow sensing and movement and adjust a body’s motion with physically and chemically sensing^33^. Most current soft robot systems require complicated assembly processes or special 3D fabrication processes^12,34^.

Some of the reported soft robots that utilize material stretchability or unique 3D structures can be fabricated using simple 3D printing with appropriate materials^35^. Such a fabrication process is suitable for on-demand fabrication and rapid prototyping. On the other hand, 2D monolithic film robot fabrication is advantageous for mass production^33,36^. Electroactive polymer (EAP) based monolithic thin film robot (MTFR) with multi layered structure^36^, and programmable magnetized film robot using cast methods^37,38^ were fabricated in past. This method is highly adaptable to various material, but it’s not fully compatible with conventional 2D lithography techniques.

Here, we demonstrate a new monolithic fabrication process for large-scale production of soft film robots, which are highly compatibility with integration circuits. Integrated and miniaturized robotic structures can be fabricated using the proposed process without complicated wiring or assembly processes; only a top-down process with lithography or simple machining for an electro thermal robotic film is required. These film robots could be applied to kinetic flexible printed circuit (FPC) substrates and combined with various electrical devices or IC chips such as LED chip. This method has the potential to serve as a basic technique for new “kinetic” electrical circuits such as next-generation body–machine interfaces, active mechanical sensors, embedded self-inspection systems for electrical circuits, and disposable medical device end-effectors. We named this technique as kinetic electronics as a natural extension of flexible electronics.

**Figure 1:**
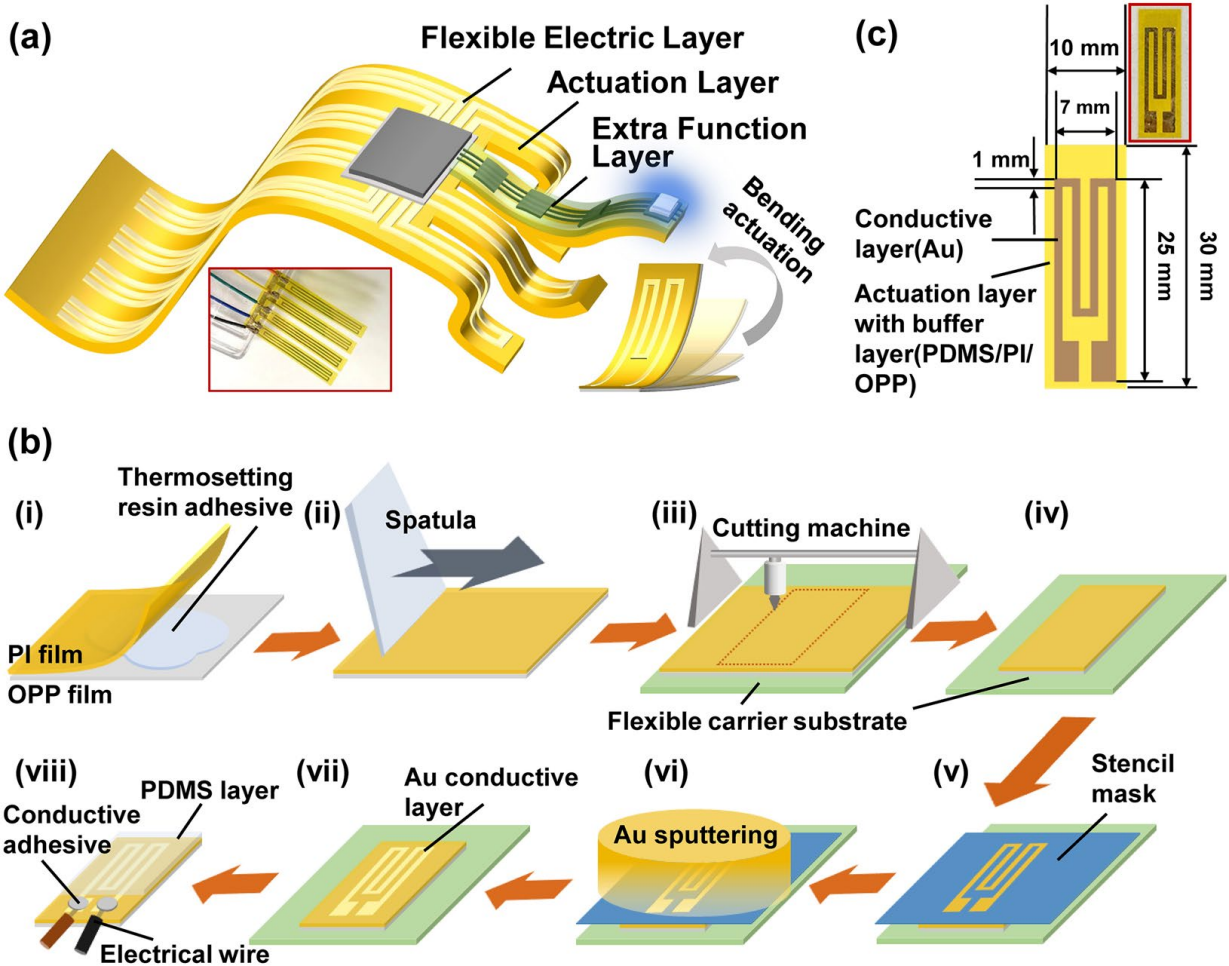
Concept of layered, flexible, film microrobot fabrication. (**a**) Schematics of layered film soft robot structure. (**b**) Schematic of monolithic fabrication process of layered film microrobot: (i, ii) Bonding of base films for forming bimorph actuator film; (iii) Cutting the outline of a film robot; (iv–vi) Arrangement of stenciled mask to robot outline and Au deposition by sputtering for electrical layer patterning; and (vii, viii) PDMS mechanical buffer layer coating and wiring. (**c**) Single-joint single-finger film robot design and photograph (inset).

The concept of a monolithically processed soft film robot is shown in Fig. 1a. The film robot consisted of layers with different functions, including a flexible electric actuator layer and an extra functional layer. The actuator layer drives the motion of the film and other mechanical motion. The extra functional layer can be used for implementing devices or structures, such as heater, sensor, illumination, microflow channel, bone structure. The film was temporally applied to support the substrate and then processed. In this study, a bimorph flexible film was employed as the actuator layer. The film can be used as an electrothermal actuator, an electrical film substrate, and a robot body when an electric circuit layer is formed on the film.

## Results and discussion

### Processing of actuator film for developing a microrobot

The monolithic fabrication process of a film microrobot is shown in Fig. 1b. First, the actuator film prepared by adhesive bonding [Fig. 1b(i–ii)] was applied to a cutting sheet, which worked as a flexible carrier substrate with temporary adhesive. Then, the surface of the actuator film was wiped with 99.9% ethanol. The film fixed on the cutting sheet was set to a cutting plotter (STiKA SV-8, Roland, Japan) and cut according to the designed robot outline pattern [Fig. 1b(iii)]. Next, a stenciled mask for the heater electrode pattern was prepared with the same cutting plotter with an adhesive cutting sheet. The stenciled mask was aligned manually and applied to the surface of the actuator film [Fig. 1b(iv, v)]. Then, an Au electrical layer (50 nm) was deposited to the sheet by a sputtering machine (SC-701HMCII, Sanyu electronics, Japan) [Fig. 1b(vi)]. The stenciled mask was removed, and the film robot was taken off from the carrier substrate sheet [Fig. 1b(vii)]. Then, poly(dimethylsiloxane) (10%; PDMS; KE-1300 T, Shin-Etsu Chemical, Japan) diluted with hexane (085-00416, Fujifilm Wako Pure Chemical Corporation, Japan) was coated by using the dip coating method [Fig. 1b(viii)]. The surface was dried and cured at room temperature overnight to form a thin PDMS layer (4 µm). The PDMS layer was formed to prevent mechanical damage of the Au layer; it also worked as a mechanical buffer layer to other stacked layers owing to its low Young’s modulus (2.6 MPa^39^). The mechanical effect of the PDMS film on deformation of the actuator film calculated by product of Young’s modulus and thickness are 0.02% and 0.03% compared with the PI film and the OPP film used in this experiments respectively, so it can be negligible. Finally, the contact pad of the heater electrode was connected to an electrical wire by a conductive adhesive (DOTITE D-362, Fujikura Kasei, Japan) to connect the driving circuit. Figure 1c shows the design and photograph of a single-joint single-finger film microrobot formed by this process with oriented polypropylene (OPP)/polyimide (PI) bimorph actuator film.

### Evaluation of thermal deformation of bimorph actuator films

To evaluate thermal deformation of bimorph actuator films, OPP, PI , copy paper (Paper), and aluminum foil (Al) were employed as the base films to fabricate five types of actuator films (PI/OPP, Paper/OPP, Al/OPP, Paper/PI, Al/PI). The prepared five types of bimorph actuator films exhibited bending deformation to a constant curvature at a constant corresponding temperature. They returned to their original curvature at room temperature after cooling down. The curvature 1/r of a strip-shaped thin bimorph actuator can be calculated considering the mechanics of material modeling^40,41^. It can be expressed as a function of temperature through the following equation.

1 $$\frac{1}{r} = \frac{{6E_{1} E_{2} t_{1} t_{2} \left( {t_{1} + t_{2} } \right)\left( {\alpha_{1} - \alpha_{2} } \right)\Delta T}}{{\left( {E_{1} t_{1}^{2} } \right)^{2} + \left( {E_{2} t_{2}^{2} } \right)^{2} + 2E_{1} E_{2} t_{1} t_{2} \left( {2t_{1}^{2} + 3t_{1} t_{2} + 2t_{2}^{2} } \right)}}$$

Here, Δ*T* is the temperature difference from original (bonding) temperature, *α* is coefficient of thermal expansion,* t* is thickness of the film, and *E* is the Young’s modulus.

**Figure 2:**
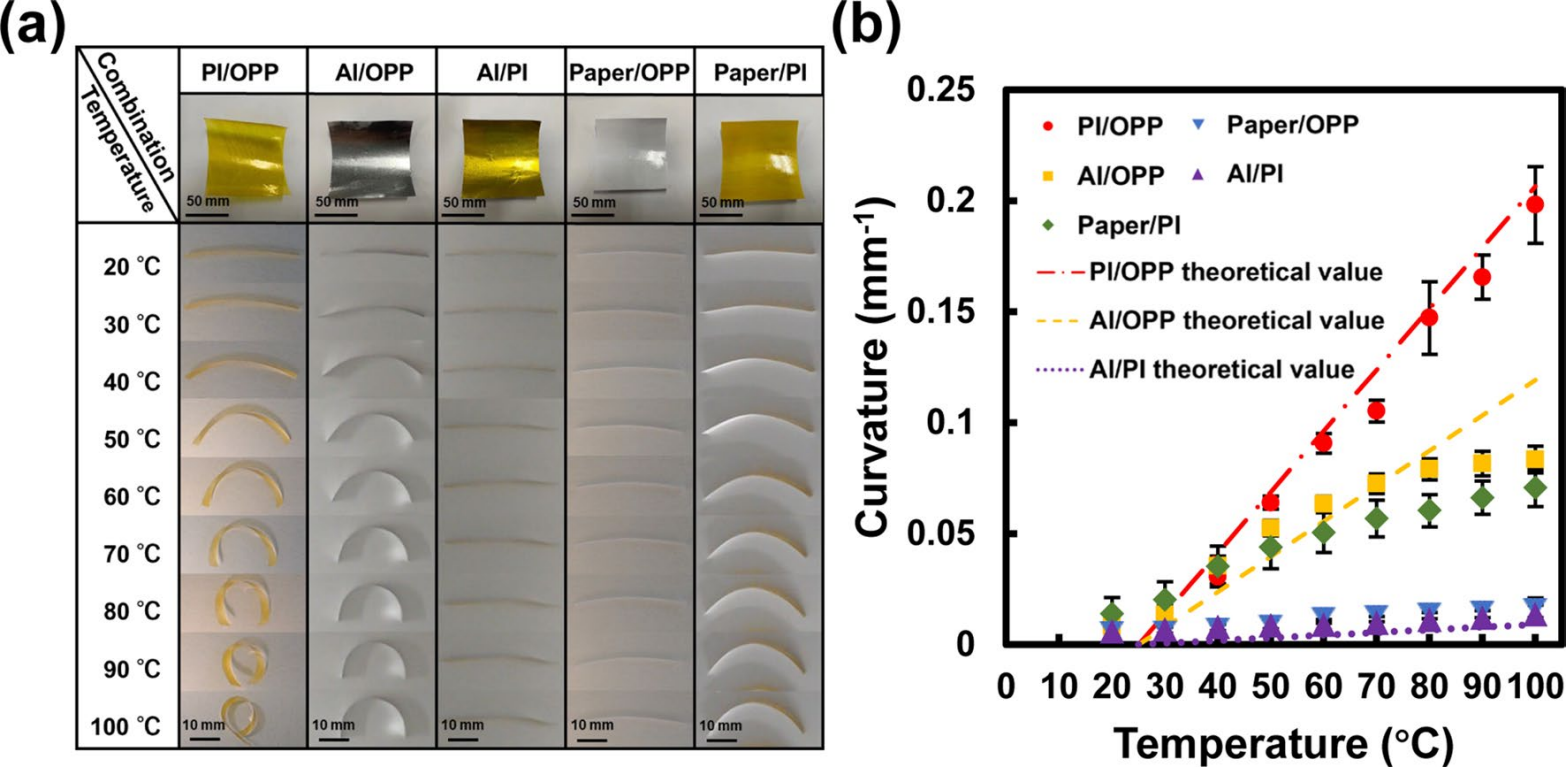
Evaluation of thermal deformation of bimorph actuator films. (**a**) Photographs of fabricated bimorph actuator films and their deformations. (**b**) Relationship between curvature and temperature. Bars represent standard deviation (n = 5).

Photographs of the deformation of the films are shown in Fig. 2a. Figure 2b shows the theoretical and experimental changes in the curvature of each film at different temperatures. The initial curvature of each specimen at room temperature (25 °C) was approximately 0.01 mm^−1^; however, the curvature at the maximum temperature (100 °C in this experiment) varied greatly from 0.01 to 0.2 mm^−1^. PI/OPP, Al/PI, and Paper/OPP films showed a linear change in their curvature with the temperature range in this experiment, and the curvature values of PI/OPP and Al/PI were in good agreement with the theoretical values. In contrast, the rate of curvature change of PI/Paper and Al/OPP decreased from approximately 50 °C. This may be due to the instable adhesion between the base films. The high-performance PI/OPP bimorph actuator film was used as the actuation layer in the film microrobot experiments.

### Characteristics of robotic film actuation

To evaluate the basic actuation characteristics of the film microrobots, a single-finger robot shown in Fig. 1c was used. For each test, one new robot film was used, and the experiment was performed five times. The maximum bending curvature of the robot film with 5 V driving voltage was 0.15 mm^−1^; and in periodic actuation, the amplitude of bending curvature (lowest curvature to highest curvature) was 0.045 mm^−1^ and 0.023 mm^−1^ at period T = 8 s and T = 4 s, respectively. The actuator performance of the film robot was comparable to resent advanced electrothermal bimorph actuators that employ advanced processes or materials to achieve high performance. Some of the reported remarkable devices are reported such as elastomer based actuator having silver nano wire (AgNW) network embedded below the surface of PDMS (curvature: 0.26 mm^−1^, driving voltage: 4.5 V, cyclic driving period: T = 120 s)^29^, and an actuator consisting of super aligned carbon nano tube (SACNT)/OPP (curvature: 0.10 mm^−1^, driving voltage: 5 V, cyclic driving period: T = 25 s)^42^. Recently, an actuator based on AgNW film of the interpenetrating structure of two different diameters and low-density polyethylene (LLDPE) shows large bending actuation with low voltage (curvature: 0.88 mm^−1^, driving voltage: 1 V, cyclic driving period: T = 20 s)^43^ was reported. Heater electrodes of most of these actuators are uniform layers of the laminated structure. Thus, electrode pattern and body structure of the actuators depend on each other. On the other hand, our robot was fabricated using top-down processing of independent thermal bimorph actuator film. Thus, the electrode pattern design, including resistance of each driving heater, is not restricted by body structure. This leads to simple robot designing, and allow to easily use the robotic film fabricated by this process as kinetic FPC.

Detailed evaluation of the properties of our device property is described below.

### Relationship between deformation and applied power

**Figure 3:**
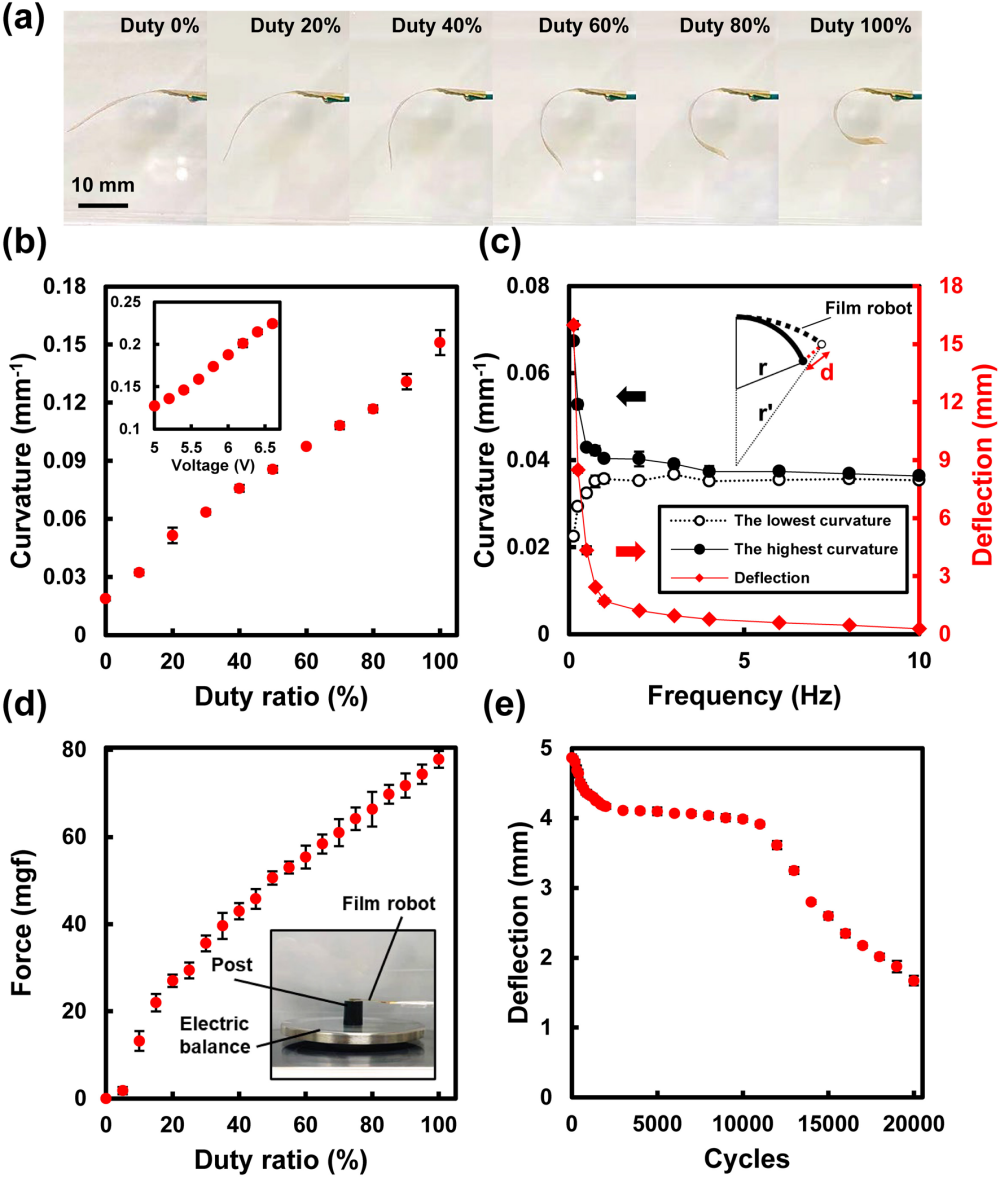
Actuation characteristics with single-finger film microrobot. (**a**) Photograph of robot actuation with different duty ratios with a 5 V PWM power supply. (**b**) Dependency of curvature on supplied power (5 V PWM) and on DC voltage change at 100% duty ratio (inset). (**c**) Frequency response of actuator deformation shown as minimum and maximum curvature on the amplitude (left axis) and tip deflection (right axis). (**d**) Generated force at zero deflection point. and photograph of measurement setup (inset). (**e**) Repetitive actuation durability of the robot on 20,000 bending motions. Bars represent standard deviation (n = 5).

To determine the relationship between the film microrobot deformation and applied power, a constant power was applied to the robot film, and the curvature was measured when the film reached a steady state of heat dissipation and heat supply following the start of thermal deformation and stopped when a constant deformation was observed. The drive voltage was fixed at 5 V and the power supply to the heater was controlled using pulse width modulation (PWM; Fig. 3a). The changes in curvature are shown in Fig. 3b. The curvature of the film robot showed a linear relationship with the power supplied with good reproducibility (the worst case: relative STD 4.7% at 100% duty ratio). In the same way, the curvature of the robot was measured using a DC drive in the range of 5–6.6 V (Fig. 3b inset); the curvature also showed a linear relationship with the supplied power.

### Evaluation of speed of film robot actuation

To evaluate the robot speed, the frequency response of the robot deformation caused by a DC pulse signal was measured. A 5 V DC pulse with a pulse width of 25% of the cycle time was applied and the change in curvature and the movement of the robot tip were measured in the range of 0.125–10 Hz. Figure 3c and Movie S1 shows the maximum and minimum values of the robot curvature in each cycle. The deflection of robot tip *d* is also shown. At the lowest frequency in this experiment (0.125 Hz), the difference in the robot curvature between the maximum and minimum values was approximately 0.045 mm^−1^. However, the amount of change rapidly decreased as the frequency increased. Considering that the curvature of the electrothermally driven bimorph is a function of temperature change, it is believed that the shorter the heat-dissipation cycle, the smaller is the change in temperature. In general, electrothermally driven bimorphs can be heated rapidly using a high voltage; however, the rate of heat dissipation is determined by the difference in temperature from the atmosphere, which limits their operating speed^27^.

### Relationship between generated force and applied power

The force generated when the displacement of the robot tip was zero was measured. Figure 3d shows the force generated by the robot when driven with a 5 V PWM power supply at duty ratios of 0–100%. The force generated by the robot increased monotonically with increasing supplied power. The maximum force generated was 78 mgf, which is 5.7 times greater than the weight of the robot film (14 mg).

In a low power range, no visible shape deformation of the robot was observed. However, with an increase in the supplied power, the tip of the film in the contact area between the robot and the plastic post was deformed, and the contact area increased. This deformation is thought to be the cause of the decreasing rate of generated force with supplied power.

### Repetitive actuation durability of robot

To evaluate the durability of the robot, high-count repetitive bending actuation was performed. Here, a 5 V DC pulse with a pulse width of 25% of the cycle time was applied, and 20,000 repetitive motions were recorded with a video camera. The five-pulse average of the deflection of the robot tip is shown in Fig. 3e. During the initial 2000 repetitive motions, the amplitude decreased monotonically. Then, it stabilized at a constant value until 10,000 repetitions. After that, the amplitudes rapidly decreased. At this time, the initial heater resistance was 150 Ω; the final resistance after 20,000 repetitions was 149 Ω, which is nearly the same as the initial resistance. The decrease in the amplitude may be owing to the aging process during the initial stage, such as microscale adhesion damage to the robot film. Extension of the small crack from initial defect of bonding suggests accumulation of the damage (Fig. S1). Enhancement of adhesion strength by using liquid primer coating or plasma treatment^44^ for bonding process of actuator film could be effective to prevent the damage and improve the actuation lifetime. The results indicated that this simple film could be used for precise bending operation for approximately 8000 cycles with appropriate aging treatment.

### Demonstration of film robots with multiple degree-of-freedom actuation

**Figure 4:**
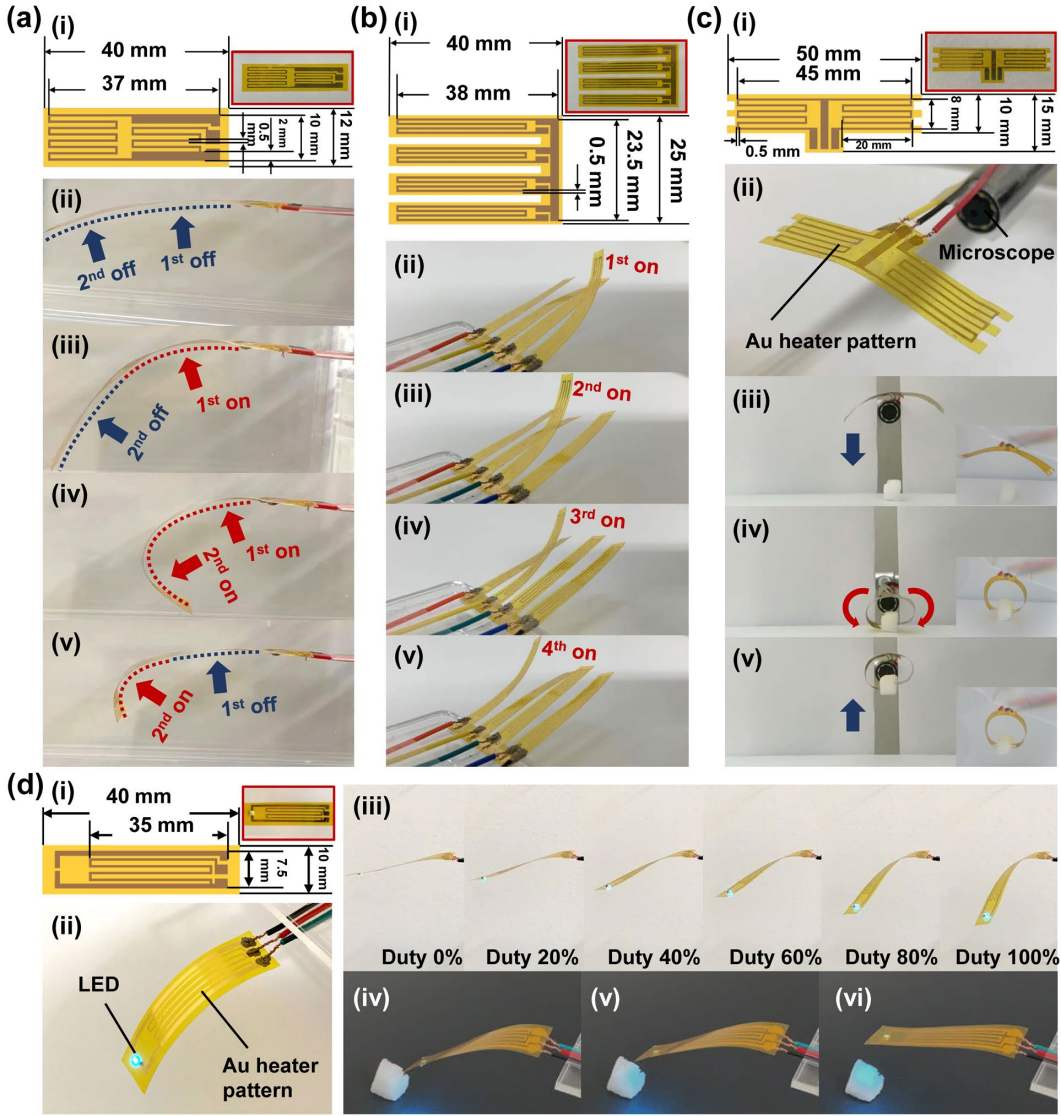
Multi-degree-of-freedom (DOF) film microrobots. (**a**) A two-joint single-finger robot: (i) Design and photograph; (ii) Initial state of the finger robot; and (iii–v) In-plane motion by 2 DOF control. (**b**) A four-fingered array robot: (i) Design and photograph; (ii–v) Sequential independent finger actuations. (**c**) A two-fingered gripper robot: (i) Design and photograph; (ii) Gripper mounted on a microscope probe; and (iii–v) Gripping of styrofoam cube by 2 DOF control. (Inset) view from mounted microscope. (**d**) Design (i) and photograph (ii) of a mobile illuminator robot; (iii) Illumination positions with different duty ratios using a 5 V PWM power supply; (iv–vi) Illumination of an object from different lighting positioning.

To demonstrate the proposed film robot fabrication process, we fabricated multi-degree-of-freedom robots with various electrode patterns and shapes. Robots can be fabricated by simply changing the design patterns in the process shown in Fig. 1b. The design and operation photographs of the robots fabricated in this study are shown in Fig. 4. The two-joint single-fingered robot (Fig. 4a, Movie S2) with a series of heater electrodes can move the robot tip to a point on a plane by heating two heaters independently. The finger array, which has four independent fingers (Fig. 4b, Movie S3), can be driven up and down to perform contact operations on flat objects. It can be applied to large-scale multiplexing of cell colony pickers and reagent addition probes used in biological experiments^45^. A gripper with a pair of opposing fingers (Fig. 4c, Movie S4) can grasp and release an object precisely by manipulating the left and right fingers independently. It was used to grasp a styrofoam cube weighing 6 mg. The gripper was fixed to the tip of an optical microscope probe, so that the robot could be operated while observing the object, as shown in the microscope image in Fig. 4c[iii–v (inset)]. A mobile illuminator film robot was also fabricated (Fig. 4d, Movie S5). A blue color surface-mounted chip LED (SMLM12ABC7W1, Rohm semiconductor, Japan) with the dimensions of 2 × 1.25 mm^2^ was manually mounted on the robot film using a conductive adhesive. The robot illuminated an object from different positions during robot motion [Fig. 4c(ii–v)]. The blinking pattern and light intensity were controlled independently of robot motion. The illumination function is an example of extra functional layer device. Such a robot can be used for the examination of part failure inside device housing using the circuit or for cavitas detection^46,47^ in human body by wearable moving sensors.

## Conclusion

We developed a monolithic soft microrobot fabrication process for the mass production of low-cost robots with a complicated structure by simple 2D processing of a robotic actuator film. We investigated several materials for the base bimorph actuator film and evaluated the characteristics of the PI/OPP film, which showed particularly excellent characteristics. The developed film microrobot is remarkably light (with a density of only 45 µg/mm^2^), and it can be processed easily. The film robot can be driven by a low voltage of 5 V using PWM control. Our experiments indicated that it is possible to perform precise position control repeatedly with a relative standard deviation of 4.7% in the worst case. In particular, the film robot showed stable repetitive actuation performance (greater than 8000 actuations). To demonstrate the potential and simplicity of this process, we fabricated two-joint, multi-fingered, and gripper robots with multiple degrees of freedom and a mobile illuminator film robot. Each could be precisely manipulated with multiple degrees of freedom, thus indicating that the proposed process was simple and could easily be used to form a variety of robots through patterning. Additionally, both the OPP and PI sides of the film can be used to form electrodes by sputtering or other conventional lithography techniques. Electronic circuits or sensors can be then integrated to these films. The films could be used as a kinetic flexible printed circuit for various electronic circuits, computers, and probe chips for biological and chemical experiments; these applications can be termed as “Kinetic electronics”.

## Materials and methods

### Fabrication of bimorph flexible actuator films

For the actuation layer of the film microrobot, we prepared a bimorph flexible actuator film by lamination bonding with four base films having different thermal and mechanical properties. Oriented polypropylene (OPP) (SO25-1, Hattori, Japan), polyimide (PI) (Kapton 50H, Dupont, USA), copy paper (Paper) (WC901PET, APPJ, Indonesia), and aluminum foil (Nippaku foil, Mitsubishi-aluminum, Japan) were employed as the base films to fabricate five types of actuator films (PI/OPP, Paper/OPP, Al/OPP, Paper/PI, Al/PI). The properties related to the bimorph actuator of the base film are listed in Table 1. To fabricate the actuator film, each base film was first cut to 100 mm squares and rubbed on both sides by a clean room-grade paper wipe (Bemcot PS2, Asahi-kasei, Japan) with 99.9% ethanol. Then, the bonding side of the base film was washed with flowing ethanol and dried completely by placing it in pure atmosphere for 10 min. Next, a commercial adhesive for difficult-to-adhere polymers (SU05141, KONISHI, Japan) 10% diluted with acetone was applied to the bonding side of the film. The second film was carefully overlapped from the edges of the first film to form a pair of films. A spatula was used on one side of the film to squeeze the excess adhesive and air bubbles between the films. Then, the pair of films were sandwiched between flat glass plates weighing 3 kg. The pair of films were then placed on a flat hot plate set at the bonding temperature (25 °C for all experiments described in this paper) for 2 h. The fabricated actuator film flattened at the bonding temperature.

**Table 1:** Thermal and mechanical properties of the film material for bimorph actuator films.

Material	Thickness (µm)	Young's modulus (GPa)	Coefficient of thermal expansion (1/°C)
PI^48^	12.5	3.5	2.0 × 10^−5^
OPP^49^	20	1.9	8.0 × 10^−5^
Al	12	69	2.4 × 10^−5^
Paper	90	N.A	N.A

### Thermal deformation of bimorph actuator films

For each combination of bimorph actuator films, specimens of size 40 mm × 5 mm were cut to establish the relationship between curvature and temperature. The specimens were placed on a hot plate, and a video camera (C980GR, Logitech, Swiss land) was set vertically at a top-view angle to observe the deformation of each film from 20 to 100 °C. The curvatures were calculated from the captured photographs.

### Driving system for microrobot

The developed film microrobot was driven by a microcontroller (Arduino nano, Gravitech, USA) via a custom-made solid-state relay circuit. To eliminate the variation in actuation caused by the heater and film geometry, PWM was used for robot control. PWM power signals with an amplitude of 5–10 V were used for each robot finger driven with a tuned duty ratio. In this experiment, a desktop DC stabilization power supply (AD-8724D, AND, China) and a DC power source on a breadboard were used as power sources. All robot driving and manipulation experiments were conducted using this setup. The film robot was fixed on a polystyrene plate, and robot actuation was recorded using a video camera. All experiments were conducted at approximately 20 °C in the laboratory.

### Generated force and robot power measurements

An electric balance (TW323N, SHIMADZU, Japan) was used to measure the force and power generated by the robot. The electric balance was set to zero after a plastic post was placed on its weighing dish. The accuracy of the electric balance was ± 0.001 g. A downward lateral force was applied to the post by bending actuation of the robot finger. Force measurements began when at the vertical position that was just in contact with the robot finger to the post without a load.

## Supplementary Information

Supplementary Information 1.

Supplementary Information 2.

Supplementary Information 3.

Supplementary Information 4.

Supplementary Information 5.

Supplementary Information 6.

## Data Availability

The data that support the findings of this study are available from the corresponding author upon reasonable request.
