# Supplementary material for: Monolithic processing of a layered flexible robotic actuator film for kinetic electronics
Source: Sci Rep. 2021 Oct 8;11:20015. doi: 10.1038/s41598-021-99500-9 (PMC8501038; doi:10.1038/s41598-021-99500-9)
Supplement: Supplementary file 1 — Supplementary Information 1. [file 41598_2021_99500_MOESM1_ESM.docx]

**Supplementary Information for**

**“Kinetic electronics: Monolithic processing of a layered flexible robotic actuator film for simple film microrobot fabrication”**

Shiyi Zhang, Joseph Wang*, Kenshi Hayashi and Fumihiro Sassa*

* Joseph Wang and Fumihiro Sassa

Email: sassa@ed.kyushu-u.ac.jp

This file includes:

Figure S1

Legends for Movies S1 to S5

Other supplementary materials for this manuscript include the following:

Movies S1 to S5


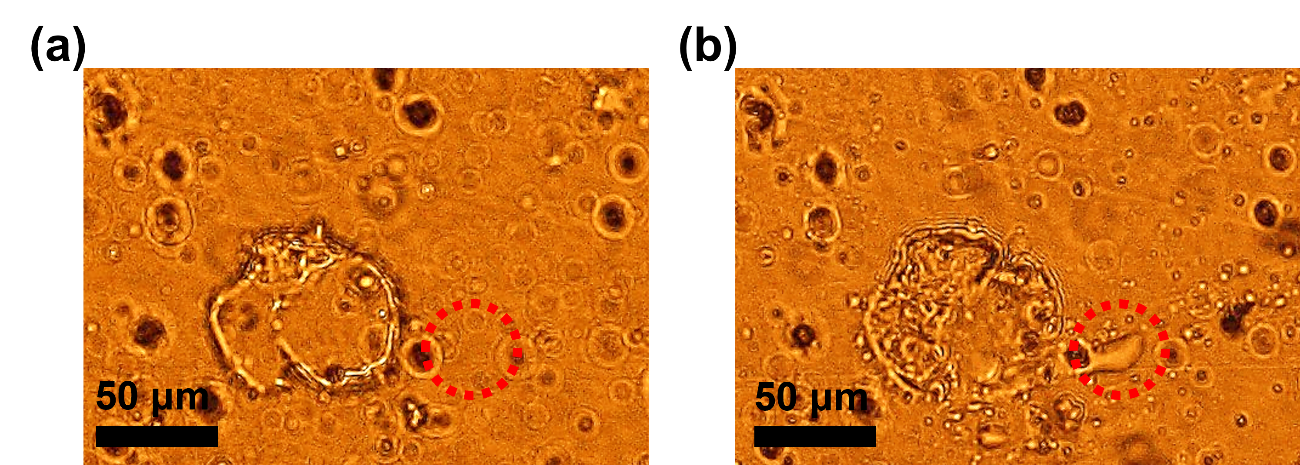


**Figure S1.** Extending of crack in PI film and OPP film bonding by repetitive bending actuation. Red dotted circle shows a place of crack extended from (a) initial state to (b) after 20,000 of repetitive actuation.

**Movie S1 (separate file).** This video shows the cyclical bending actions of a single-finger film robot at different frequencies (0.125, 0.25, 0.5, 1, 5, and 10 Hz) under the adjustment of pulse width modulation (PWM). The experimental conditions for the front and back half are 50% and 100% duty ratios, respectively, driven by the 5 V PWM signal.

**Movie S2 (separate file).** This video shows the actuation of a two-joint single-fingered film robot bending the front and rear parts separately and together. The sequence of actions in a single cycle is set as follows: the rear is bent, while the front maintains its original state; the front and rear parts are bent together; the rear part is bent back and the front half is maintained in the bent position; and the front and rear parts are bent back. The robot was driven by a 5 V PWM signal.

**Movie S3 (separate file)**. This video shows the independent actuation of a four-fingered array robot at different bending frequencies (0.25, 0.4, 2, and 4 Hz). The robot was driven by a 6 V PWM signal. At the end of the video, the results are shown together for comparison.

**Movie S4 (separate file).** This video shows the two-fingered gripper robot actuation and the manipulation of a styrofoam cube with a mass of 6 mg. The robot was driven by a 5 V PWM signal The video is divided into three parts: the first part shows the front view, the second part shows the microscope view, and third part is the 2-DOF actuation of the suspended robot.

**Movie S5 (separate file).** This video shows the actuation of the illuminator film robot. The robot illuminates a white object from different positions with continuous lighting and with blinking light. The robot was driven by a 5 V PWM signal for robot actuation and a 3.3 V PWM signal provided power for the LED.
